# Ultrastructure expansion microscopy in *Trypanosoma brucei*

**DOI:** 10.1098/rsob.210132

**Published:** 2021-10-13

**Authors:** Ana Kalichava, Torsten Ochsenreiter

**Affiliations:** ^1^ Institute of Cell Biology, University of Bern, Switzerland; ^2^ Graduate School for Cellular and Biomedical Sciences, University of Bern, Switzerland

**Keywords:** *T. brucei*, expansion microscopy, centriole, kDNA, cell structure, super resolution

## Abstract

The recently developed ultrastructure expansion microscopy (U-ExM) technique allows us to increase the spatial resolution within a cell or tissue for microscopic imaging through the physical expansion of the sample. In this study, we validate the use of U-ExM in *Trypanosoma brucei* measuring the expansion factors of several different compartments/organelles and thus verify the isotropic expansion of the cell. We furthermore demonstrate the use of this sample preparation protocol for future studies by visualizing the nucleus and kDNA, as well as proteins of the cytoskeleton, the basal body, the mitochondrion and the endoplasmic reticulum. Lastly, we discuss the challenges and opportunities of U-ExM.

## Graphical abstract

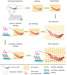

## 1. Introduction

*Trypanosoma brucei* is a unicellular flagellated protozoan parasite and the causative agent of human African sleeping sickness and Nagana in cattle. The highly polarized parasite cell body is about 25 µm in length and is shaped by the subpellicular microtubule corset [1,2]. The *T. brucei* cell contains at least eight single-copy organelles, including the (i) flagellum that emanates from the (ii) flagellar pocket in the posterior region of the cell and is attached along the entire cell body by the (iii) flagellar attachment zone (FAZ) [3]. The axoneme of the flagellum nucleates at the (iv) basal body, which aside from the well-conserved microtubule organization also associates with the (v) microtubule quartet that has an orientation (posterior to anterior) opposite of the microtubule corset [4]. Additional single-copy organelles are the (vi) Golgi and the (vii) mitochondrion [5,6] with its (viii) single mitochondrial genome named the kinetoplast DNA (kDNA). The kDNA is the feature structure of the Kinetoplastea and in *T. brucei* is organized in a complex structure of approximately 5000 interlocked mini- and approximately 25 maxicircles [7,8]. Replication and segregation of the mitochondrial genome has been studied in some detail, and it is thought that more than 100 proteins are likely to be involved in these two processes [9,10]. In recent years, a number of studies have started to deconstruct the segregation machinery (tripartite attachment complex, TAC) connecting the basal body of the flagellum to the kDNA.

**Figure 1.**
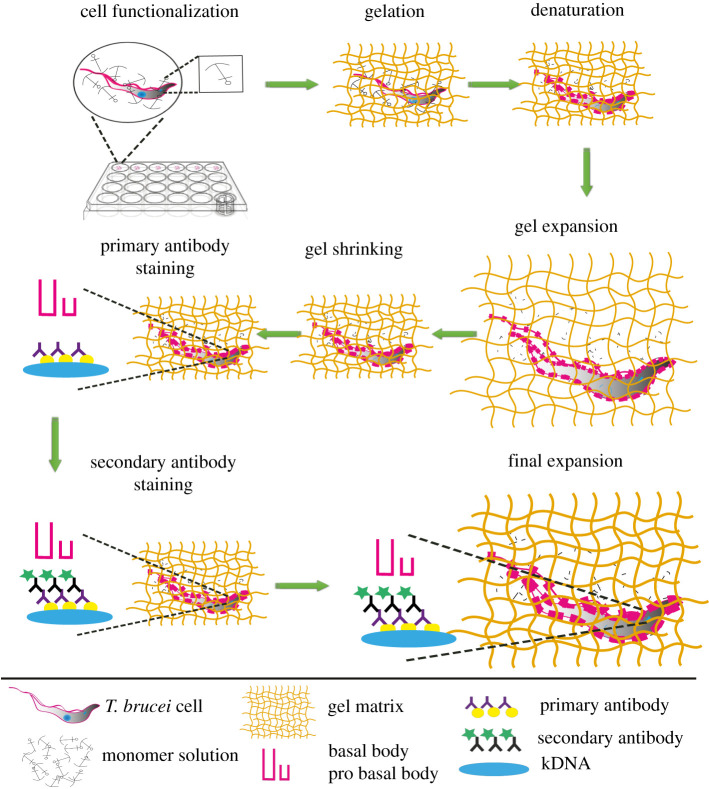
Schematic representation of the ultrastructure expansion microscopy concept in *T. brucei.* First, cells were settled at room temperature on coverslips. Then, the coverslips were incubated in a 24-well plate filled with a solution of formaldehyde and acrylamide in PBS. Cells were subsequently prepared for gelation by carefully transferring coverslips into a gel solution supplemented with APS and TEMED. After gelation, the cell components were denatured using heat and SDS, which was followed by first round of expansion and gel shrinkage in PBS. Then, cells were stained with primary and secondary antibodies followed by dialysing in water for the final expansion.

Expansion microscopy is based on a series of chemical steps to physically magnify the cell and visualize ultrastructures by optical microscopy (for details see [11–13]; figure 1; table 1 summarizes details of antibodies used in this study). In the first step, the precursor molecules formaldehyde and acrylamide are introduced to functionalize the cellular components allowing the subsequent linkage of swellable polyelectrolyte gel (sodium acrylate). The gel formation is accomplished analogously to polyacrylamide gels by using oxidizing reagents like tetramethylethylenediamine and ammonium persulfate (APS). After the gel is formed, the cell contents are denatured and thus homogenized through the use of sodium dodecyl sulfate (SDS) and heat. At this point, the sample is ready for the application of standard immunofluorescence techniques. Expansion microscopy has been applied to a wide range of organisms and scientific questions, including membrane biology in bacteria, neuronal development in fish and cell structure of protozoan parasites, to name just a few [11,14–16].

**Table 1.** Antibodies used in this study.

primary antibody	target	source/reference	dilution for U-ExM	dilution for IF
TAC102 (mouse)	TAC102	[29]	1 : 500	1 : 5000
20H5 (mouse)	basal body and pro-basal body	Millipore Sigma (04–1624)	1 : 500	1 : 2000
SAS6 (rabbit)	basal body and pro-basal body	[23]	1 : 500	1 : 2000
YL1/2 (rat)	tyrosinated tubulin	[30]	1 : 2000	1 : 50 000
Bip (rabbit)	endoplasmic reticulum	[27]	1 : 500	1 : 2000
alpha-tubulin (guinea pig)	alpha-tubulin	Geneva Antibody Facility	1 : 500	1 : 2000

secondary antibody	source	dilution for U-ExM	dilution for IF	used for
Alexa Fluor 594 (mouse)	Sigma SAB4600180	1 : 500	1 : 2000	alpha-tubulin
Oregon Green 488 (rabbit)	Invitrogen A32744	1 : 500	1 : 2000	20H5, TAC102
Alexa Fluor 488 (rat)	Thermofisher O-11038	1 : 500	1 : 2000	SAS6
Alexa Fluor 647 (guinea pig)	Thermofisher A-11006	1 : 500	1 : 2000	YL1/2

We now provide data to validate this new sample preparation technique in *T. brucei* and give an overview of the opportunities and challenges with ultrastructure expansion microscopy (U-ExM) in the protozoan model system.

## 2. Results and discussion

**Figure 2.**
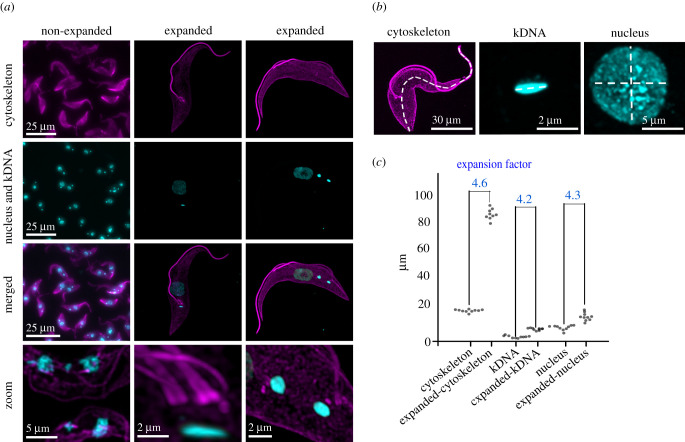
Isotropicity and the expansion factor. (*a*) Non-expanded and expanded PCF cells stained with α-tubulin (magenta; Alexa Fluor 647), kDNA and nuclear DNA (cyan; DAPI). Imagery was deconvolved using Huygens Professional and visualized using Imaris. (*b*,*c*) Magnified views and measurements of the cytoskeleton length, kDNA length and nucleus diameter in non-expanded and expanded cells were done in ImageJ. Expansion factor was calculated as the ratio between non-expanded and expanded cell compartments.

The use of ultrastructure expansion microscopy was first demonstrated in Kinetoplastea localizing a component of the mitochondrial genome segregation machinery (TAP110) relative to its interaction partner TAC102 [17]. In that study, the isotropicity was measured using the basal body, the kDNA and the nucleus as references. The expansion factors of those cellular compartments/complexes varied less than 10% between 3.61 and 3.86. In the current study, we were able to increase the expansion factor to 4.2–4.6 as measured by the expansion of the kDNA, nucleus and cytoskeleton maintaining the variation at about 10% (figure 2*a–c*). Aside from the measurements of cellular compartments, the isotropicity of the expansion becomes obvious in the maintenance of the overall cell shape when compared to the non-expanded trypanosomes (figure 2*a,b*).

**Figure 3.**
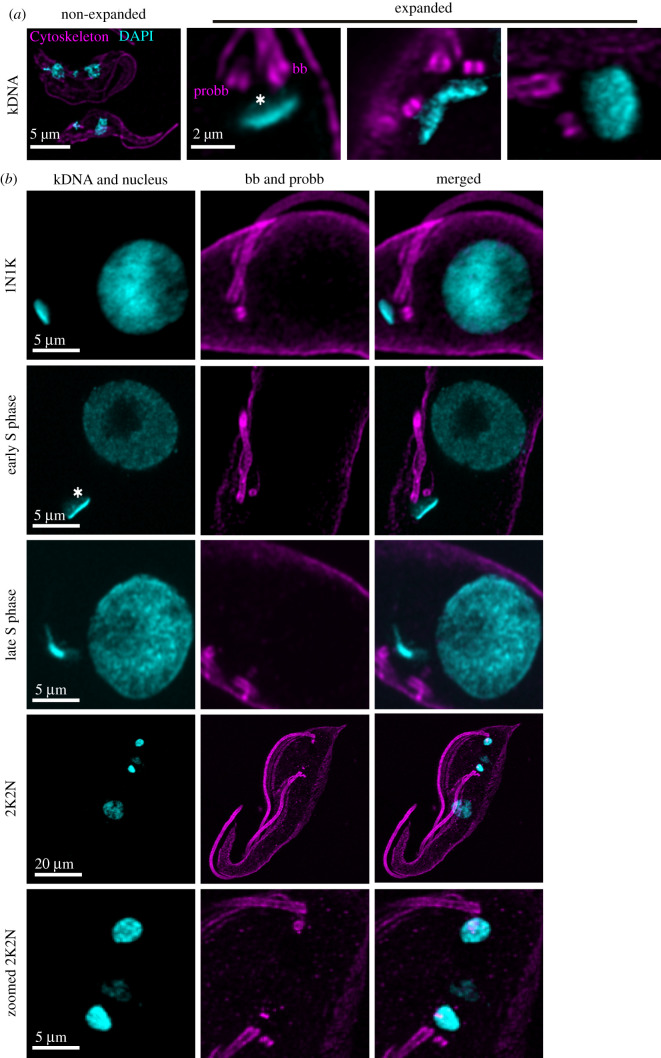
Representative confocal images of kDNA during different stages of the cell cycle. (*a*) Comparative confocal images of expanded and non-expanded *T. brucei* kDNA (DAPI, cyan). (*b*) Representative images of the kDNA and basal bodies during different kDNA replication steps. DNA stained with DAPI (cyan); cytoskeleton stained with a monoclonal alpha-tubulin antibody (magenta; Alexa Fluor 647). Asterisk marks the ‘cloudy’ part of the kDNA. Imagery was deconvolved using Huygens Professional and visualized using Imaris.

The ability to image DNA in the nucleus and the mitochondrion of expanded cells will in future allow the precise localization of expressed genes, centromeric sequences or overall chromatin architecture. Thus, we evaluated the use of the minor groove AT-rich DNA binder DAPI in U-ExM in *T. brucei*. The mitochondrial DNA of *T. brucei* and for most Kinetoplastea is organized in a complex structure of interlocked minicircles and maxicircles. Depending on the imaging angle, this results in the DAPI stain to appear as a homogeneous cylinder or ‘disc-like’ structure (figure 3*a*,*b*) [18]. In the kDNA, the minicircles are parallel to each other and stretched out maximally thereby defining the height of the cylinder. As a consequence of this organization, the kDNA cylinder can be isotropically expanded in its diameter but not in its height (figure 3*a*). With U-ExM, we can visualize individual regions within the kDNA disc that have previously only been detected by electron microscopy. This includes the inner unilateral filament region, a DNA containing domain between the kDNA disc and the inner mitochondrial membrane (figure 3*b*) [19]. If the ‘cloudy’ DAPI signal at the basal body proximal face of the kDNA disc is made up of minicircle replication intermediates as could be predicted by the current replication model [9] or potentially maxicircle segments remains to be investigated (figure 3*a*,*b*, asterisk). Furthermore, during replication and prior to segregation, the kinetoplast assumes a conformation that from the side resembles a V. In this conformation, the DAPI signal seems strongly concentrated in the middle between the two newly developing kDNAs (late kDNA replication phase), which is likely a combination of the imaging angle and the replication of maxicircles that potentially occurs at the interphase of the two replicating kDNAs [20] (figure 3*b*)**.**

**Figure 4.**
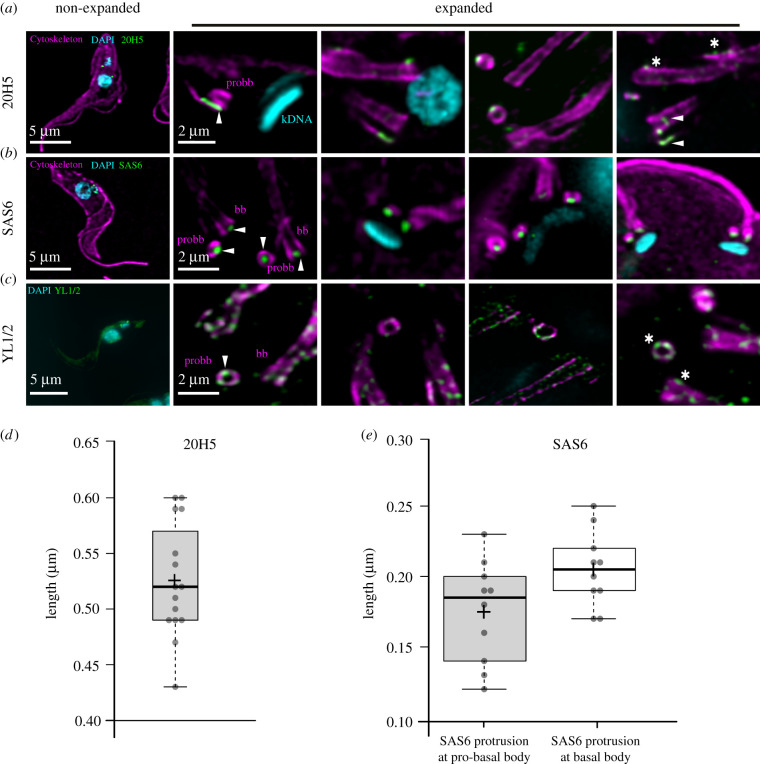
Representative confocal images of basal body associated structures in U-ExM. (*a*) Centrin stained with anti-centrin antibody 20H5 (green), anti-α-tubulin antibody (magenta), kDNA and nucleus with DAPI (cyan). Arrowheads mark the centrin signal at the maturing basal body and microtubule quartet. Asterisk represents centrin signal at the old basal body and bilobe structure. (*b*) Anti-SAS6 antibody (green) localization at the basal body and pro-basal body (magenta). (*c*) Tyrosinated tubulin distribution (YL1/2, green) at the bb and probb (magenta). (*d*) Boxplot quantification of the length of 20H5 signal at the pro-basal body. (*e*) Quantification of SAS6 protrusion at the pro-basal body (the left graph) and basal body (the right graph). Imagery was deconvolved using Huygens Professional and visualized using Imaris.

In *T. brucei*, the cytoskeleton is dominated by microtubules that can be visualized by an alpha-tubulin antibody. Aside from the subpellicular microtubules, the antibody also allows detection of the entire axoneme from the base of the flagellum to its tip, revealing its continuous attachment to the cell body as previously described [21] (figure 2*a*). By using higher zoom factors, we can visualize the pro- and mature basal bodies with their associated microtubule quartet in proximity to the kDNA (figure 2*a*). The basal body is the central organizer of the cell cycle specifically involved in the duplication of the Golgi and the mitochondrion, including its singular genome [4,6,22]. Although the precise number remains unknown, probably more than 100 proteins are involved in the biogenesis and structure of the basal body, all concentrated in a small area. Thus, precise localization of the individual components will be key for understanding their role in basal body biogenesis. Centrins, for example, are calcium-binding proteins that are highly conserved in all centrosomes. The monoclonal antibody 20H5 that was raised against the centrin in Chlamydomonas binds to centrin 1 and 2 at the basal body and the bilobe region in *T. brucei*, [6] (figure 4*a*). Using U-ExM, we can now show that centrin1/2 are found in expanded cells at the mature basal body, and a region of about 500 nm between the maturing basal body and the associated growing microtubule quartet (figure 4*a*, arrowhead; quantification in figure 4*d*). To a lesser degree, there is also a signal at the old basal body (figure 4*a*, asterisk) and at the bilobe (figure 4*a*, asterisk). A second very prominent protein of the basal body is SAS6, a structural component of the cartwheel, that in trypanosomes is present at the pro- and the mature basal body [23] (figure 4*b*). U-ExM confirms that similar to the recent data from cryo-tomography SAS6 is in the centre of the cartwheel and protrudes at the proximal end of the basal body [24]. We measured the protrusion to be 170 and 210 nm at the pro-basal body and mature basal body, respectively (figure 4*e*). In non-expanded cells, this would translate to 40–50 nm that the cartwheel would protrude from the proximal end of the basal body (figure 4*e*). Another commonly used reagent to label the basal body is the antibody YL1/2 that binds to tyrosinated α-tubulin and also cross-reacts with the basal body protein TbRP2 [25,26]. With U-ExM and at high dilutions of the primary antibody, we find tyrosinated tubulin to be more or less equally distributed at the old and new basal body. At these concentrations, the previously described cross-reactivity of YL1/2 with TbRP2 cannot be detected (figure 4*c*, asterisks).

**Figure 5.**
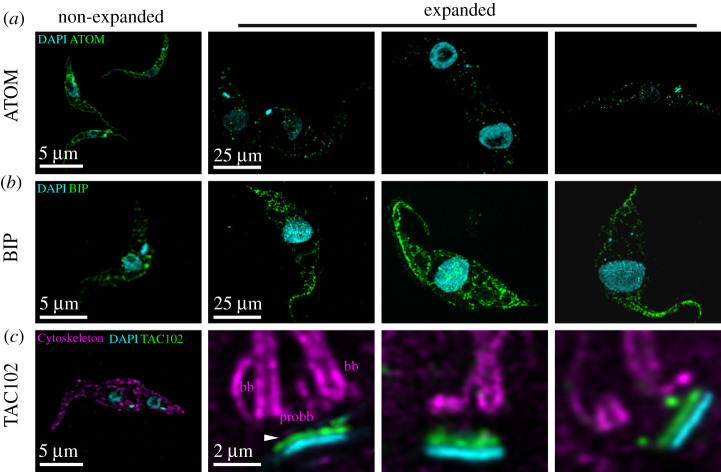
Expansion microscopy targeting different compartments of *T. brucei.* (*a*) Mitochondrial protein complexes (anti-ATOM antibody, green) distribution in *T. brucei*. (*b*) BIP (anti-BIP antibody, green) distribution in *T. brucei*. (*c*) TAC protein (anti-TAC102 monoclonal antibody, green) localization at the kDNA. Imagery was deconvolved using Huygens Professional and visualized using Imaris.

One question that we wished to address was whether membrane-bound organelles could be analysed using U-ExM in *T. brucei*. To test this, we localized the archaic protein translocase of the outer membrane (ATOM), which is part of the mitochondrial protein import machinery in the outer mitochondrial membrane, TAC102 which is a TAC protein inside the mitochondrion and the luminal ER component binding protein (BiP) [27–29]. In regular confocal microscopy, ATOM is continuously distributed throughout the entire mitochondrial membrane. In expansion microscopy, the distribution of the protein is visible in several hundred individual spots per cell, suggesting individual complexes can be visualized (figure 5*a*). The ER-resident protein BiP on the other hand seems to be more continuously distributed throughout the ER lumen even in U-ExM (figure 5*b*), which is in good agreement with its function as a chaperone. TAC102 that was previously shown to reside inside the mitochondrion in proximity to the kDNA disc can be visualized using a monoclonal antibody (figure 5*c*)

### 2.1. Ultrastructure expansion microscopy challenges and opportunities

U-ExM offers several technical advantages for visualizing ultrastructures, but it also faces significant challenges. One of the main chemicals used in U-ExM is sodium acrylate. Its quality varies from batch to batch, and impurities negatively affect polymerization and ultimately the expansion factor. Another key challenge in expansion microscopy is the imaging process. The transparent aqueous gel starts shrinking after some time due to laser power and the evaporation of water out of the gel, which results in shifted images. Furthermore, sample storage is not as simple as for immunofluorescence slides. While immunofluorescence slides can be kept at four degrees for a long period of time, gel quality drops rapidly. It should also be noted that U-ExM requires significantly more antibody than typical immunofluorescence staining. Also, we have experienced potentially due to the denaturation of epitopes that not all antibodies that work in regular immunofluorescence microscopy can be used in U-ExM. U-ExM enables super-resolution imaging through the improvement of axial and lateral effective resolution by an isotropic increase in the distance between individual molecules to be analysed. Correspondingly, expansion by a factor of four increases resolution from around 200 nm to less than 50 nm using regular confocal microscopy and significantly further by super-resolution techniques. In future, this will allow us to precisely visualize and characterize biomolecular structures that are in close proximity to each other such as components within the basal body and nuclear pore complexes. In contrast with other high-resolution imaging techniques, U-ExM is accessible without the need of special equipment. In combination with protease treatment, the technique will likely allow improved intra-molecular resolution leading to *in situ* structure analysis, while using established reagents. Many antibodies and stains used for regular immunofluorescence microscopy show good results also in U-ExM. Furthermore, by tuning the sample functionalization steps and increasing the expansion factor, resolution in the single-digit nanometer range seems feasible. In summary, U-ExM has developed into a very powerful tool bridging the gap between light and electron microscopy.

## 3. Material and methods

### 3.1. *Trypanosoma brucei* cell culture conditions

Procyclic form (PCF, 29–13) asynchronous *T. brucei* cells were cultured in semi-defined medium-79 (SDM-79) supplemented with 10% FCS, 15 µg ml^−1^ geneticin and 25 µg ml^−1^ hygromycin at 27°C.

### 3.2. Immunofluorescence analyses

One million cells were washed twice with 200 μl phosphate-buffered saline (PBS; 137 mM NaCl, 2.7 mM KCl, 10 mM Na_2_HPO_4_, and 1.8 mM KH_2_PO_4_. PBS) and centrifuged for 3 min at 1800*g*, then resuspended in 100 µl of PBS and settled for 15 min at room temperature on coverslips. Then, the excess liquid is removed with tissue paper. Then, 200 µl of 4% paraformaldehyde (PFA; Sigma P6148) were overlaid on the cells adhered on coverslips and incubated at room temperature for 5 min. Coverslips were then washed 3 times with 200 μl PBS and then permeabilized with 0.2% Triton X-100/PBS for 4 min. After blocking coverslips with PBS containing 4% BSA for 0.5 h, trypanosomes were stained in 4% BSA/PBS with primary antibodies: guinea pig tubulin detecting α-tubulin (AA345, Geneva Antibody Facility) 1 : 2000; YL1/2 antibody detecting tyrosinated tubulin as present in the basal body [30] 1 : 100 000; monoclonal mouse TAC102 antibody (Trikin *et al*. [29]) 1 : 2000; polyclonal rabbit ATOM (gift from Andre Schneider) 1 : 2000; mouse 20H5 (Millipore 04–1624) 1 : 2000; polyclonal rabbit SAS6 (gift from Ziyin Li)1 : 2000; rabbit p67 and rabbit BIP (gift from James Bangs) 1 : 2000. After thoroughly washing with 200 µl PBS, cells were incubated with 100 µl secondary antibodies Alexa Fluor 488 Goat-anti-Rabbit IgG (H + L) (Invitrogen), Alexa Fluor 594 Goat-anti-Mouse IgG (H + L) (Molecular Probes), Alexa Fluor 647 Goat-anti-Rat IgG (H + L) (Life Technologies), anti-guinea pig 594 (Abcam 150188) and 647 (Abcam 150187) diluted all 1 : 10 000 in 4% BSA/PBS for 1 h. The cells were counterstained with DAPI before mounting with Gold ProLong Gold antifade reagent (Molecular Probes D1306). Fluorescent optical images were taken on a Leica TCS SP8 using a 63 × 1.4 NA oil objective. LAS X software (Leica Microsystems), Huygens Professionals and ImageJ were used to analyse the images.

### 3.3. Ultrastructure expansion microscopy

Two million cells were washed twice with 200 µl PBS and centrifuged for 3 min at 1800*g*, then resuspended in 150 µl of PBS and settled for 20 min at room temperature on poly-D-lysine functionalized coverslips (12 mm, Menzel-Glaser). Coverslips were transferred into a 24-well plate filled with a solution of 0.7% Formaldehyde (FA, 36.5–38%, Sigma) with 0.15% or 1% acrylamide (AA, 40%, Sigma) in PBS and incubated for five hours at 37°C. Cells were then prepared for gelation by carefully putting coverslips (cells facing down to the monomer solution) into 35 µl of monomer solution (AK Scientific 7446-81-3) 10% (wt/wt) AA, 0.1% (wt/wt) N,N′-methylenebisacrylamide (BIS, SIGMA) in PBS supplemented with 0.5% APS and 0.5% tetramethylethylendiamine (TEMED) on parafilm in a pre-cooled humid chamber. Gelation proceeded for 5 mins on ice, and then samples were incubated at 37°C in the dark for 1 h. Coverslips with gels were then transferred into a six-well plate filled with denaturation buffer (200 mM SDS, 200 mM NaCl and 50 mM Tris in ultrapure water, pH 9) for 15 min at room temperature. Gels were then detached from the coverslips with tweezers and moved into a 1.5 ml Eppendorf centrifuge tube filled with denaturation buffer and incubated at 95°C for 1 h and 30 min. After denaturation, gels were placed in beakers filled with deionized water for the first round of expansion. Water was exchanged at least twice every 30 min at room temperature, and then gels were incubated overnight in deionized water. Next day, gels were washed two times for 30 min in PBS and subsequently incubated on a shaker (gentle) with primary antibody diluted as follows: guinea pig tubulin detecting α-tubulin (AA345, Geneva Antibody Facility) 1 : 500; YL1/2 antibody detecting tyrosinated tubulin as present in the basal body [30] 1 : 100 000; monoclonal mouse TAC102 antibody [29] 1 : 1000; polyclonal rabbit ATOM (gift from Andre Schneider) 1 : 500; 20H5 (Millipore 04-1624) 1 : 500; polyclonal rabbit SAS6 (gift from Ziyin Li) 1 : 1000; rabbit p67 and rabbit BIP (gift from James Bangs) 1 : 500; all antibodies were diluted in 2% PBS/BSA and gels were incubated for 3 h at 37°C. Gels were then washed in PBST three times for 10 min while gently shaking and subsequently incubated with secondary antibodies Alexa Fluor 488 Goat-anti-Rabbit IgG (H + L) (Invitrogen), Alexa Fluor 594 Goat-anti-Mouse IgG (H + L) (Molecular Probes), Alexa Fluor 647 Goat-anti-Rat IgG (H + L) (Life Technologies), anti-guinea pig 594 (Abcam 150188) and 647 (Abcam 150187) all 1 : 1000 and 4′,6-diamidino-2-phenylindole (DAPI, Thermofisher D1306) 1 : 5000. All antibodies were diluted in 2% PBS/BSA and gels were incubated for approximately 3 h at 37°C. Gels were then washed in PBST three times for 10 min while gently shaking and finally placed in beakers filled with deionized water for expansion. Water was exchanged at least twice every 30 min before gels were incubated in deionized water overnight. Gel expanded between 4.1 × and 4.4 × depending on SA purity.

### 3.4. Mounting and image acquisition

After the final expansion, gel size was measured with a caliper to calculate the fold expansion. The gel was then cut with a razor blade in pieces that fit in a 36 mm metallic chamber for imaging. The piece of gel was then mounted on a 24 mm round poly-D-lysine functionalized coverslip, already inserted in the metallic chamber and gently pressed with a brush to ensure adherence of the gel to the coverslip. Confocal microscopy was performed on a Leica TCS SP8 using a 63x 1.4 NA oil objective, with the following parameters: z step size at 0.3 μm interval with a pixel size of 35 nm. LAS X software (Leica Microsystems), Huygens Professionals and ImageJ were used to analyse the images.

### 3.5. Calculation of expansion from ultrastructure expansion microscopy

The expansion factor was determined by comparing the ratio of expanded cell length, kDNA and nucleus to non-expanded cell length, kDNA and nucleus. For unexpanded kDNA and nucleus measurements, *n* = 20 cells from immunofluorescence imagery were analysed. The length of the kDNA was determined by measuring the maximum length of the kDNA observed in each cell. The diameter of the nucleus was determined by measuring the widest diameter observed in each cell. The length of the cell structure was determined by measuring the cell length from anterior to posterior end in each cell.

## Data accessibility

Data are all in the manuscript. Original imagery is available on request.
